# Microplastic Contamination in Snow from Western Italian Alps

**DOI:** 10.3390/ijerph18020768

**Published:** 2021-01-18

**Authors:** Marco Parolini, Diego Antonioli, Franco Borgogno, Maria Cristina Gibellino, Jacopo Fresta, Carlo Albonico, Beatrice De Felice, Susanna Canuto, Donatella Concedi, Alessandra Romani, Emanuela Rosio, Valentina Gianotti, Michele Laus, Roberto Ambrosini, Roberto Cavallo

**Affiliations:** 1 Department of Environmental Science and Policy, University of Milan, via Celoria 26, 20133 Milan, Italy; beatrice.defelice@unimi.it (B.D.F.); roberto.ambrosini@unimi.it (R.A.); 2 Department of Sciences and Technological Innovation, University of Piemonte Orientale, Via T. Michel 11, 15121 Alessandria, Italy; diego.antonioli@uniupo.it (D.A.); Valentina.gianotti@uniupo.it (V.G.); Michele.laus@uniupo.it (M.L.); 3 European Research Institute, via Pinelli 24/d, 10144 Torino, Italy; frankieborgogno@gmail.com (F.B.); c.susanna@eri.net.in (S.C.); 4 Laboratorio Agenzia Regionale Protezione Ambiente della Valle d’Aosta Loc. La Maladière n. 48, Saint Christophe, 11020 Aosta, Italy; m.gibellino@arpa.vda.it (M.C.G.); c.albonico@arpa.vda.it (C.A.); d.concedi@arpa.vda.it (D.C.); al.romani@arpa.vda.it (A.R.); 5 ERICA Soc. Coop., via Santa Margherita, 26, 12051 Cuneo, Italy; jacopo.fresta@cooperica.it (J.F.); roberto.cavallo@cooperica.it (R.C.); 6 AICA International Association for Environmental Communication, 12051 Cuneo, Italy; emanuela.rosio@cooperica.it

**Keywords:** MPs, pollution, Aosta Valley, high-mountain, Italian Alps

## Abstract

Recent studies have documented the presence of microplastics (MPs) in remote areas, including soils or sediments collected in mountain and glacier environments, but information on their presence in snow is scant. The present study aimed at exploring the presence of MPs in residual snow collected in four locations of the Aosta Valley (Western Italian Alps), with different accessibility and human presence. Overall, the µ-FTIR analyses confirmed the presence of 18 MPs in snow, 7 (39%) items were fibres, while 11 (61%) were fragments. Polyethylene (PE; 7 MPs) was the main polymer, followed by polyethylene terephthalate (PET; 3 MPs), high density PE (HDPE; 3 MPs), polyester (2 MPs), while only 1 MP made by low density PE, polypropylene and polyurethane were found. The mean (± SE) concentration of MPs in snow ranged between 0.39 ± 0.39 MPs/L and 4.91 ± 2.48 MPs/L, with a mean of 2.32 ± 0.96 MPs/L for the sampling locations. The concentration of MPs did not statistically differ among locations. Our results suggest that MPs presence in high-mountain ecosystems might depend on deposition through atmospheric precipitations or local sources due to human activities. For these reasons, policies aiming at reducing plastic use and dispersal in mountain areas may be effective in preventing local MP contamination.

## 1. Introduction

In the last decade, the environmental contamination by microplastics (MPs), plastic items ranging between 1 μm and 5 mm in size [1], has raised increasing attention in the society and the scientific community because of ethical and aesthetical considerations, as well as because of concerns related to their potential impacts on ecological systems and human health [2]. A large number of studies have documented the presence of MPs in atmosphere, many different domains of marine ecosystems, freshwaters, soil, and biota, confirming that these contaminants represent a serious environmental concern [3,4,5]. Whilst the presence of MPs has been pointed out in both anthropic and natural ecosystems worldwide, recent surveys demonstrated the occurrence of MPs also in the so-called remote areas. For instance, MPs items have been found in remote marine ecosystems, such as the deep sea, Southern Oceans, Arctic, and Antarctica [6,7]. Moreover, MPs have been isolated from pelagic water and shoreline debris from high-mountain lakes [8,9], floodplain soils of Alpine valleys [10] and recently from the supraglacial debris of the Forni Glacier (Italian Alps), where 74.4 ± 28.3 (SE) items/kg of dry sediment were measured [11], an amount similar to those found in medium-contaminated European coastal sediments [6].

The origin of MPs in high-mountain ecosystems can be due to the breakout or degradation of large plastic items, which were abandoned involuntarily or deliberately by humans, in smaller sizes through physical-chemical or biological processes [12], as well as by the wear of clothes and equipment [11]. However, recent studies demonstrated that atmospheric transport represents the main pathway of MPs to remote areas, including high-mountain ones [13]. Accordingly, the presence of MPs in remote, sparsely inhabited mountain areas of the French Pyrenees, far from emission sources, has been attributed to atmospheric transport [14]. Although the origin of MPs in the atmosphere are not well understood [15], studies performed in China, Iran, and France have demonstrated that precipitations could represent the main pathway of larger MPs to the ground [16,17,18], while conversely larger counts of smaller MPs (and nanoparticles) have been reported during dry deposition [19,20].

In high-mountain ecosystems, snow is the main scavenger for diverse airborne impurities [21]. The presence of MPs in snow from Arctic, Swiss, and Bavarian Alps, as well as from the city of Bremen (Germany), has been demonstrated, suggesting that atmospheric transport and wet deposition can be considered as pathways for MPs contamination in remote areas [22]. In fact, this study has demonstrated the presence of high MPs concentrations in snow from all the study areas, whereby the concentrations of MPs measured in Arctic snow were lower than those found in that from European locations [22]. In addition, a recent study has shown the presence of MPs in snow collected around a lake in Carnic Alps [23]. Despite these findings, the information on MPs contamination in snow from mountain ecosystems is still limited. Thus, the aim of the present study was to explore the presence of MPs in residual snow collected in late summer 2019 in four high-mountain (>2500 m a.s.l.) locations of the Aosta Valley (Western Italian Alps).

## 2. Materials and Methods

### 2.1. Sampling Locations

**Figure 1:**
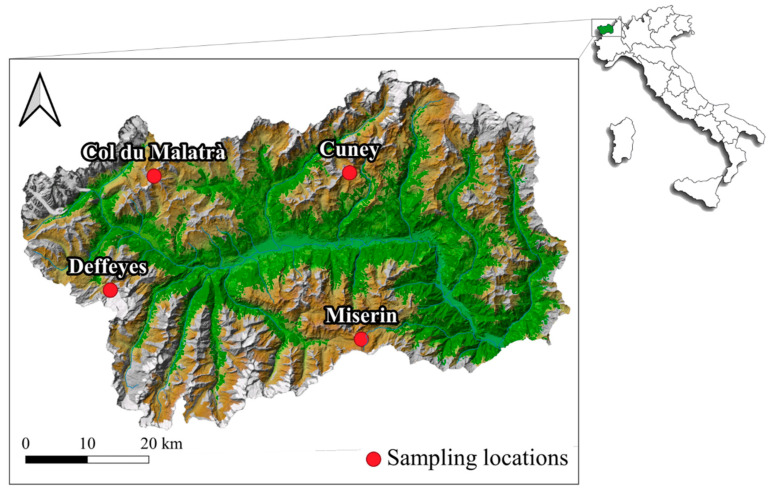
Map of the sampling area (Aosta Valley, Western Italian Alps). Red dots indicate the sampling locations.

Residual snow was collected between 7th and 11th of September 2019 in four locations on the path of the Tor des Géants^®^ trail running race (Aosta Valley, Western Italy; Figure 1). Sampling was performed at the same time as the race. Sporting events, in particular trail running, represent one of the useful collection tools in environmental research, also for their popularization potential. For instance, in 2019, a research of MPs in the Po river had been conducted thanks to the Keep Clean And Run event with considerable media coverage [24] (https://keepcleanandrun.com/).

Sampling locations were identified according to the degree of isolation from human impact, in terms of the ease to access the location, which were identified as low (easy to be reached) to high (difficult to be reached). In detail, the sampling was performed close to the Cuney hut (hereafter Cuney), the Miserin hut (hereafter Miserin) and the Deffeyes hut (hereafter Deffeyes), as well as at Col du Malatrà (hereafter Malatrà). Characteristics of sampling locations are reported in Table 1.

Miserin is easy to reach because it is accessible by car, Cuney and Deffeyes are moderately easy to reach by 2.5–3 h walk, while Col du Malatrà, the pass that separates the Ferret valley from the Gran San Bernardo valley, can be considered as hard to reach by >4 h walk.

### 2.2. Snow Sampling

Snow was not sampled on the path of the race but in neighbouring areas with no direct access for athletes of tourists. Snow sampling was performed by appropriately trained operators with a stainless steel spoon, scraping the surface of snow accumulation, and transferred to 2 L glass jars. Snow surface was scraped to remove airborne material that could interfere with the analytical procedure of MP isolation. We collected an integrated sample of residual snow that has been deposited over the year. Both the spoon and the glassware were previously washed with acetone. Three independent samples per each location were collected (identified in Table 1 with the Roman numeral I, II, and III). The opening of the jar was wrapped with tinfoil to avoid external contamination. To prevent contamination by the operators, they were dressed in 100% cotton clothes and the sampling was performed downwind. Samples were transported to the laboratory and were maintained at 4 °C in the dark until the isolation of MPs.

### 2.3. Isolation and Characterization of Microplastics

All the glassware, stainless forceps and pins used during the analytical procedure were previously washed with acetone and then with ultrapure water filtered on cellulose filters (StonyLab, pore size 1 µm; Ø = 47 mm) and wrapped in tinfoil until analyses to avoid laboratory contamination. A total of 12 snow samples were collected. The volume of water (i.e., melted snow) of each sample was determined by transferring the water to a graduated cylinder (Table 1).

The water sample (500 mL) was then transferred to a 1 L beaker and an appropriate amount of sodium chloride was added in order to saturate the solution (density = 1.2 g/cm^3^ corresponding to 365 g NaCl/Lj) [25]. When the volume of water samples exceeded 500 mL, we replicated the procedure until processing all the sample. Solubilisation of sodium chloride was performed by 30 min stirring using a glass-covered magnetic stirring rod. The solution was then transferred in 600 mL separation funnel. The beaker in which the salt solution was prepared was washed three times with ca. 20 mL of a NaCl solution (density = 1.2 g/cm^3^) previously filtered on cellulose filters (StonyLab, pore size 1 µm; Ø = 47 mm) to collect any residual item. Washing aliquots were added to the sample within the separation funnel and the solution was allowed to settle overnight. The solution at the bottom of the separation funnel was removed, while the upper phase of the solution (100 mL) was added with 100 mL of 30% hydrogen peroxide filtered solution to remove organic matter overnight. The solution was then transferred to a glass separation flask, where it was filtered on cellulose filters (StonyLab, pore size 1 µm; Ø = 47 mm) through a water-jet pump. The separation funnel was washed three times with filtered ultrapure water (20 mL) and the washing aliquots were filtered on the same filter used for the sample. The filter was placed in glass petri dish (Ø = 50 mm) and dried into a desiccator for 48 h. Five samples (i.e., batch) of melted snow were processed contemporarily. A procedural blank was run with each batch of snow samples, processing 500 mL of filtered ultrapure water as described above.

Preliminary visual inspection (according to shape and colour of items) of the filters was performed under a Leica EZ4 W stereomicroscope to check for the presence of putative MPs. Items identified as putative MPs during the preliminary visual inspection were transferred individually to a silver metal membrane filter (Sterlitech, pore size 0.8 µm; Ø = 13 mm) with stainless pins. A picture of each single item transferred to the filter was captured to allow the measurement of size (expressed as the maximum length of item) with the image processing package Fiji freeware software [26]. In order to check for potential aerial contamination of the laboratory, we placed a cellulose filter on a tinfoil next to the equipment we used during the whole analytical procedure, as reported by Winkler et al. [27]. Such filter was analysed as described above. No item <5 mm was found either in procedural blanks or on filters used to check for aerial contamination of the laboratory.

Fourier Transformed Infrared (FTIR) microscopy (µ-FTIR) was performed using a Nicolet iN10 MX Infrared Imaging Microscope (Thermo Scientific, Waltham, MA, USA) to characterize the polymeric composition of the isolated items. The characterization was performed in reflection mode in a wavenumber range of 4000–650 cm^−1^. The instrument was controlled by OMNIC^TM^ Picta software (Thermo Scientific, Waltham, MA, USA). A total of 256 scans were acquired for each spectrum, with a spectral resolution of 4 cm^−1^. The detection and identification limit of items for μ-FTIR instrument was 10 μm. A polymer was considered as such when the matching with the respective polymer in the library was >70%. For polymer identification the following libraries were used: HR Aldrich Polymers, HR Coatings Technology, HR Hummel Polymer and Additives, HR Industrial Coatings, HR Polymer Additives and Plasticizers, HR Rubber Compounding Materials, HR Spectra Polymers and Plasticizers, Hummel Polymer sample Library, and Polymer Laminate Films.

### 2.4. Statistical Analysis

The presence of statistically significant differences in the concentration of MPs isolated from residual snow collected at the four locations in Val d’Aosta was investigated by the non-parametric Kruskal-Wallis test because the application of the Kolmogorov-Smirnov test revealed that data were not normally distributed. Statistical analysis was performed using R statistical software (version 4.0.2; R Core Team, Vienna, Austria).

## 3. Results

Overall, regardless of the sampling location, a total of 40 putative MPs were isolated from melted snow, 27 (68%) of which were fibers and 13 (22%) were fragments or particles of other shapes. The snow samples collected at Deffeyes (16 items) contained the highest number of putative MPs, followed by those from Miserin (11 items), Malatrà (5 items), and Cuney (8 items).

The polymer characterization performed by using the µ-FTIR, confirmed the presence of MPs only in 7 of the analyzed samples (58%). Overall, a total of 18 real MPs, accounting for the 45% (=18/40 items) of the total items isolated from snow samples, 17 (43%) cellulose fibers and 1 (2%) wool fiber were identified, while 4 (10%) items were not identified (Table 2). Focusing on MPs, 7 (39%) items were fibers, while 11 (61%) were fragments (Table 2).

The size of MPs, i.e., the maximum length of each single item, fiber, or fragment, ranged between 50 and 1910 µm, with an average (± SE) length of 339 ± 103 µm. Only 2 MPs were <100 µm, while 15 MPs were <1000 µm and 1 MP was >1000 µm. Focusing on fibers, their length ranged between 83 and 1910 µm (mean value ± SE: 543 ± 251 µm), while the length of fragments ranged between 50 and 422 µm (mean value ± SE: 223 ± 34 µm).

**Figure 2:**
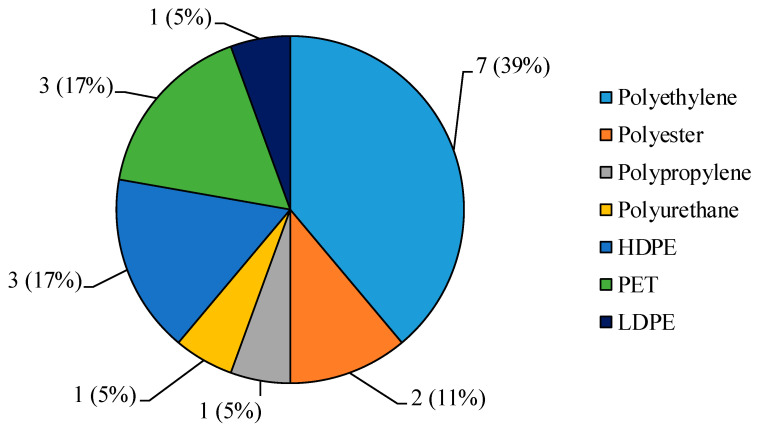
Number of microplastics (MPs) grouped per polymeric composition (% on the total amount of MPs) isolated from snow samples collected in four locations of the Aosta Valley (Western Italian Alps). HDPE = High-density Polyethylene; LDPE = Low-density Polyethylene; PET = Polyethylene terephthalate.

The main colour of MPs was white (9 MPs, 50%), followed by blue (4 MPs, 28%) and light blue (2 MPs, 11%), while pink or purple MPs were a small percentage (1 MP, 5.5% in both the cases). Polyethylene (7 MPs, 39%) was the most abundant polymer, followed by PET (3 MPs, 17%), HDPE (3 MPs, 17%) and polyester (2 MPs, 11%), while a lower contribution was given by LDPE, polypropylene, and polyurethane (1 MP per each polymer; Figure 2).

**Figure 3:**
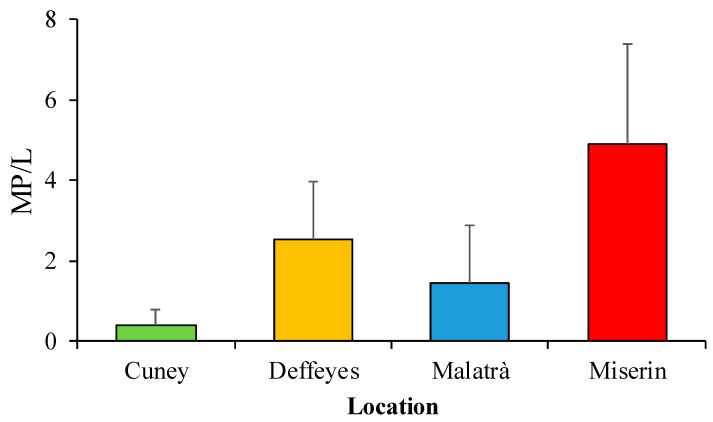
Concentration (mean ± SE) of MPs (expressed in MPs/L of melted snow; mean ± SE) in snow samples from the four locations in the Aosta Valley (Western Italian Alps).

The highest concentration of MPs (expressed as the number of MPs/L of melted snow ± SE; Figure 3) was found in snow collected at Miserin (4.91 ± 2.48), followed by Deffeyes (2.53 ± 1.45) and Malatrà (1.45 ± 1.45), while a very low concentration was found at Cuney (0.39 ± 0.39). However, the non-parametric Kruskal-Wallis test did not reveal any statistically significant difference in MPs concentration among the four locations (χ^2^ = 2.1228, degrees of freedom = 3, *p*-value = 0.5473).

## 4. Discussion

The present study provides the first evidence of MPs contamination in residual snow collected in high-mountain ecosystems on Italian Alps. Microplastic items having different size, shape, color, and polymeric composition were found in residual snow from high-mountain locations in the Aosta Valley.

Recent studies have documented that MPs can reach remote areas through several pathways. Human activities in mountain ecosystems produce a great amount of plastic garbage, which often cannot be transported down valley or is deliberately abandoned on the paths, such as food packaging, while the most of alpinist equipment is made of plastic and can be abandoned in emergency or deliberately [11]. Plastic waste experiences weathering through physical, chemical, and biological processes, which contribute to the breakage of plastic items in MPs. In addition, technical clothes for trekking, hiking, or running are made by synthetic polymers and their wear can release MPs in the environment. The Aosta Valley is crossed by several paths, which are visited all year long by tourists and athletes (e.g., for the Tor des Géants^®^ trail running race), whose clothes and equipment may release MP items. Recent findings have pointed out that MPs can be transported by wind and atmospheric currents to remote areas, where they can be deposited by dry and wet deposition [13,14,28]. In high-mountain, wet deposition represents the main pathway of MP deposition, as rain and snow precipitations can bind airborne particles and pollutants, including MPs, which are eventually deposited to the ground [29]. For instance, Allen and coauthors [14] confirmed that both MPs fibers (up to ~750 µm in length) and fragments (<300 µm in size) can be transported by air and deposited to the ground in a remote, pristine mountain catchment of French Pyrenees up to 95 km from potential emission sources. The presence of MPs in residual snow from the Aosta Valley might suggest the presence of these contaminants in the atmosphere, whose deposition to the ground could be mediated by the snow. In fact, snow has been identified to scavenge aerosol particles up to 50 times more efficiently than rain [30]. However, as MPs were measured in residual snow that was accumulated over the year, we cannot exclude that MPs may be transported by wind from local sources and not from wet deposition.

Overall, our results showed that MPs were present in all the sampling locations. The mean (± SE) concentration of MPs measured in snow (2.32 ± 0.96 MPs/L) was notably lower compared to that observed in snow samples from different geographical areas, including remote ecosystems such as the Arctic and Swiss and Bavarian Alps [22]. In detail, MPs occurred in freshly or residual snow at a mean concentration (± SE) of 9.8 × 10^3^ MPs/L (6.9 × 10^3^ MPs/L), whereby the highest concentrations were detected in freshly deposited snow collected in Bavarian Alps next to a country lane and a lake (154 × 10^3^ MPs/L) and from a backyard and next to a pedestrian path in front of the Alfred Wegener Institute campus on the Isle of Heligoland (Northern Germany, 17.6 × 10^3^ MPs/L) [22]. The highest concentrations detected in these samples could be due to human presence and activities characterizing the sampling locations. For instance, Bavarian samples were collected on a day with high car traffic, while the samples from the North Sea island of Heligoland were collected in a campus located on an isle inhabited by only ~1200 people [22]. Despite no significant differences were noted, our data showed considerable differences in MPs contamination among sites, whereby the higher number of MPs was found in snow collected from the locations easier to reach, such as Miserin and Deffeyes. These results suggest that local sources (i.e., mountain huts or paths visited by tourists) may contribute to MP amounts. Further studies with a larger sample size are needed to confirm the influence of local sources in determining the differences in MPs abundance among sampling locations.

Focusing on remote areas, the abundance of MPs in snow samples from the Aosta Valley were higher, but in the same order of magnitude, compared to that found in snow collected around a lake in a high-mountain, pristine area located in Carnic Alps (Northeastern Italy), where only a PET fiber was detected accounting for (mean ± SE) 0.11 ± 0.19 MPs/L [23]. In contrast, high concentrations of MPs were detected in snow from ice floes drifting in the Arctic Fram Strait and at different locations on Svalbard islands, with concentrations up to 14.4 × 10^3^ MPs/L and 1.1 × 10^3^ MPs/L, respectively [22]. Although the concentrations of MPs in the Arctic snow (range: 0–14.4 × 10^3^ MPs/L; mean ± SE: 1.76 ± 1.58 × 10^3^ MPs/L) were lower than those found in the snow from European locations (range 0.19 × 10^3^–154 × 10^3^ MPs/L) [22], they were on average an order of magnitude higher than those recorded in the present study. The higher MPs concentrations found in snow from Arctic and Bavarian Alps compared to Italian Alps could be due to the presence of specific local activities in the sampling locations or to the atmospheric transport of MPs emitted both in North America and Europe, as well as from high-emission regions further south [13].

Despite differences in the number of MPs, the size of fragments and the length of fibers was similar to those measured in items isolated from snow collected in the Arctic and in Swiss and Bavarian Alps [22], as well as in Carnic Alps [23]. In contrast, the polymeric composition of the MPs found in snow from Val d’Aosta differed with respect to that of Arctic locations. Whilst in our samples polyethylene was the most abundant polymer, followed by PET, HDPE and polyester, acrylates/polyurethanes/varnish/lacquer (hereafter varnish), nitrile rubber, PE, polyamide, and rubber type 3 (ethylene-propylene-diene rubber), where the main polymers detected in snow samples from Arctic [22]. The prevalence of varnish in Arctic samples can reflect the application of polymer-based varnish for protective coatings of different surfaces, such as vehicles, ships, wind turbines, aquaculture, and buildings [21]. Nitrile rubber can be originated from the abrasion of seals, O-rings, hoses, synthetic leather, cable jacketing, grommets, transmission belts in offshore oil platforms, automotive, and aeronautical industry, while rubber type 3 from the abrasion of tires or durable synthetic rubber roofing membranes used in roof construction [22]. Conversely, the polymeric fingerprint of our samples agreed that of previous studies showing that PE is the prevalent polymer found in European snow [22], as well as in atmospheric fallout of Dongguan [16] and a French household [30]. The prevalence of PE in European samples was not unexpected considering that PE, including HDPE and LDPE, accounts for the 29.7% of the European plastic demand in 2018 because of its extensive use in several things of everyday usage, mainly in food packaging [31]. The presence of PET in our samples could be due to wear and release of fibers from clothes and equipment [11]. These findings suggest different sources of MPs between Europe and the Arctic and/or the involvement of other mechanisms rather than the deposition from the atmosphere in the transport of MPs in mountain ecosystems. For instance, considering the amount of macroplastic waste (i.e., plastic items >5 mm in size) on mountain paths, mainly in areas with high tourist influx, MPs might originate from the breakage of large plastic items as a consequence of chemical, physical, or biological processes and can be dispersed by local winds.

## 5. Conclusions

Our results confirmed the presence of MPs in residual snow collected in high-mountain ecosystems. The origin of these MPs can be ascribable to atmospheric deposition through precipitations (i.e., snow or rain), which act as scavengers of airborne MPs originating from local sources and/or mid- or long-range atmospheric transport. The differences, although not significant, in the number of MPs found in the snow from the sampling locations with different accessibility should also suggest that local sources could contribute to MP contamination in mountain environments. Further studies are needed to estimate the annual deposition of MPs, in terms of number and weight, as well as to model the atmospheric transport to shed light on the potential origin of the contamination. Lastly, considering that snow melting led to the deposition of MPs to mountain soils, analyses on the ingestion and potential adverse effects on soil organisms should be a priority to assess the ecological risk of MP contamination in mountain ecosystems.

## Figures and Tables

**Table 1:** Characteristics of the four sampling locations were snow samples were collected.

Sampling Location	GPS Coordinates (Latitude, Longitude)	Elevation (m a.s.l.)	Description
Cuney	45.841770, 7.483298	2656	Reachable in about three hours only by walk
Miserin	45.601501, 7.507855	2582	Reachable by car
Deffeyes	45.672429, 6.987034	2500	Reachable in two and a half hours only by walk
Col du Malatrà	45.837024, 7.077821	2936	Reachable in >4 h only by walk

**Table 2:** Characteristics of putative MPs isolated from snow samples collected at four locations in the Aosta Valley (Western Italian Alps). Plastic polymers are reported in bold.

Location and Sample Number	Filtered Volume (mL)	ID MP	Shape	Color	Length (µm)	Polymer
Cuney I	890	MP1	Fiber	Transparent	398	Cellulose
		MP2	Fiber	Blue	133	Cellulose
		MP3	Fiber	Dark	266	n.i.
Cuney II	950	MP4	Fiber	Red	103	Cellulose
		MP5	Fiber	Blue	364	Cellulose
		MP6	Fiber	Yellow	485	Cellulose
Cuney III	850	MP7	Fiber	Blue	226	Cellulose
		MP8	Fragment	Pink	**177**	**Polyethylene**
Deffeyes I	940	MP9	Fiber	Blue	**183**	**Polyester**
		MP10	Fiber	Transparent	181	Cellulose
		MP11	Fiber	Light Blue	338	Cellulose
		MP12	Fiber	Red	290	Cellulose
		MP13	Sphere	Purple	18	n.i.
		MP14	Sphere	Purple	21	n.i.
		MP15	Fiber	Red	520	Cellulose
Deffeyes II	910	MP16	Fiber	Purple	**83**	**Polipropilene**
Deffeyes III	920	MP17	Fiber	Red	119	Cellulose
		MP18	Fiber	White	346	Cellulose
		MP19	Fiber	Red	552	Cellulose
		MP20	Fiber	Light Blue	**325**	**Polyurethane**
		MP21	Fragment	White	**422**	**HDPE**
		MP22	Fragment	White	**105**	**HDPE**
		MP23	Fragment	White	**222**	**HDPE**
		MP24	Fiber	Blue	**114**	**Polyester**
Malatrà I	270	n.d.				
Malatrà II	280	MP25	Fiber	Blue	286	Cellulose
		MP26	Fiber	Transparent	363	Wool
Malatrà III	230	MP27	Fiber	Blue	270	Cellulose
		MP28	Fiber	Red	217	n.i.
		MP29	Fragment	White	**261**	**Polyethylene**
Miserin I	600	n.d.				
Miserin II	600	MP30	Fiber	Dark	158	Cellulose
		MP31	Fiber	Blue	**1910**	**PET**
		MP32	Fiber	Blue	**281**	**PET**
		MP33	Fiber	Blue	**904**	**PET**
		MP34	Fiber	Dark	211	Cellulose
		MP35	Fragment	Light Blue	**50**	**LDPE**
Miserin III	620	MP36	Fragment	White	**413**	**Polyethylene**
		MP37	Fragment	White	**215**	**Polyethylene**
		MP38	Fragment	White	**245**	**Polyethylene**
		MP39	Fragment	White	**148**	**Polyethylene**
		MP40	Fragment	White	**192**	**Polyethylene**

ID MP = identity of each putative microplastic (MP) isolated from snow samples. Length (µm) = maximum length of each item; n.i. = the polymeric composition was not identified. No MPs were found in snow samples named Malatrà I and Miserin I (n.d. = not detected.). HDPE = High-density Polyethylene; LDPE = Low-density Polyethylene; PET = Polyethylene terephthalate.

## Data Availability

The data presented in this study are available on request from the corresponding author.
